# Is cancer risk of radiation workers larger than expected?

**DOI:** 10.1136/oem.2008.043265

**Published:** 2009-06-30

**Authors:** P Jacob, W Rühm, L Walsh, M Blettner, G Hammer, H Zeeb

**Affiliations:** 1 Hemholtz Zentrum München, Institute of Radiation Protection, Neuherberg, Germany; 2 Federal Office for Radiation Protection, Department of Radiation Protection and Health, Oberschleißheim, Germany; 3 Johannes Gutenberg – University Mainz, Institute of Medical Biostatistics, Epidemiology and Informatics, Mainz, Germany

## Abstract

Occupational exposures to ionising radiation mainly occur at low-dose rates and may accumulate effective doses of up to several hundred milligray.

The objective of the present study is to evaluate the evidence of cancer risks from such low-dose-rate, moderate-dose (LDRMD) exposures.

Our literature search for primary epidemiological studies on cancer incidence and mortality risks from LDRMD exposures included publications from 2002 to 2007, and an update of the UK National Registry for Radiation Workers study. For each (LDRMD) study we calculated the risk for the same types of cancer among the atomic bomb survivors with the same gender proportion and matched quantities for dose, mean age attained and mean age at exposure. A combined estimator of the ratio of the excess relative risk per dose from the LDRMD study to the corresponding value for the atomic bomb survivors was 1.21 (90% CI 0.51 to 1.90).

The present analysis does not confirm that the cancer risk per dose for LDRMD exposures is lower than for the atomic bomb survivors. This result challenges the cancer risk values currently assumed for occupational exposures.

Occupational and medical diagnostic exposures to ionising radiation are mainly due to Roentgen rays and gamma rays, which belong to so-called low-linear energy transfer (LET) radiation. The exposures may accumulate over a lifetime to doses of the order of 100 mGy. For example, in the 15-countries collaborative study on radiation workers in the nuclear industry, about 10% of the 407 000 study members received external doses exceeding 50 mGy, while only 0.1% received doses exceeding 500 mGy.^1^ Exposures with doses in the range of 50–500 mGy are considered here to be moderate in comparison with the high-dose groups of the atomic bomb survivors from Hiroshima and Nagasaki.

Within an hour, which is the timescale for cellular repair processes, doses from occupational and medical diagnostic exposures do not generally exceed the order of 10 mGy. Thus, these exposures occur at low-dose rate.

**What this paper adds**

- Occupational exposures to ionising radiation occur normally at low-dose rate and may sum up to moderate doses in the order of 100 mGy.
- Limits of occupational exposures are based on the assumption that cancer risk factors are lower than for the atomic bomb survivors by a factor of two.
- Twelve recent epidemiological studies on cancer after low-dose-rate, moderate-dose exposures were included in this analysis of cancer risks related to such exposures.
- The studies provide evidence that cancer risk factors for occupational exposures are not lower than for atomic bomb survivors.
- The new evidence for cancer risks should be taken into account in optimisation procedures for the use of radionuclides and ionising radiation at the work place and in medicine.

It follows that estimates of health risks, in particular of cancer risks, related low-dose-rate, moderate-dose (LDRMD) exposures are of central importance for practical radiation protection.

Current estimates of cancer risks from LDRMD exposures are mainly based on risk coefficients derived from the Japanese atomic bomb survivors, that is, from persons with acute, high-dose exposures, which are then combined with a “dose and dose-rate effectiveness factor” (DDREF).^2^ ^3^ Values for DDREF have mainly been deduced from experiments with laboratory animals and from radiobiological measurements. Specifically, the International Commission on Radiological Protection (ICRP) derived estimates of the excess cancer risk after low-dose exposures and after exposures with higher doses but low-dose rates by reducing the corresponding risk value for the atomic bomb survivors by a DDREF of 2.0.^2^ The BEIR VII Committee of the US National Research Council used a DDREF of 1.5.^3^

During the past few years, a number of epidemiological studies have been published, which provide major information on cancer risk after LDRMD exposures. The statistical power of each of these studies is not strong because of the relatively low risks of the doses involved. Therefore, the present study focuses on studies of larger groups of cancers. More specifically, studies of all cancer, all cancer excluding leukaemia, all solid cancer and all solid cancer excluding bone cancer have been included.

In the present paper, values of the excess relative risk (ERR) per dose in LDRMD studies of cancer risks from exposures to low-LET radiation are compared with those calculated for the atomic bomb survivors for the same grouping of cancer types, gender distribution, average age at exposure, average age attained and dose quantity. A combined estimator of the resulting risk ratios is calculated. Based on this risk estimator, cancer lifetime risks are assessed.

In some of the LDRMD studies, ERR-per-dose distributions include a value of zero, which would correspond to an infinite value of the DDREF. In order to avoid resulting instabilities of the calculations, the inverse DDREF value, *Q*, that is, the ratio of the ERR-per-dose value in the LDRMD study to that for the atomic bomb survivors, is calculated here.

## Methods

### Literature search

A systematic literature search for primary epidemiological studies was conducted in the PubMed database in January 2008, covering the period January 2002 to December 2007. The search terms “radiation” and “cancer” were combined with alternatives of the terms “occupation”, “work”, “personnel”, and “environmental” or “emergency”. A number of exclusion terms were specified to limit the findings to ionising radiation effects in the occupational, environmental or emergency setting. An initial selection of 714 papers was identified. The PubMed search was augmented by a manual search for references, by which a paper on Chernobyl emergency and clean-up workers^4^ and a paper on Oak Ridge National Laboratory (ORNL) workers^5^ were identified. Further, stimulated by a suggestion of a reviewer, a recent study on the UK National Registry for Radiation Workers^6^ was included in the analysis, because of its outstanding importance. Results without inclusion of this study are also reported below.

The initial selection was then restricted to cohort and case-control studies and epidemiological reviews, which left 123 papers. Further eliminations were made of studies on exposures to alpha radiation (because most of the occupational exposures are due to external radiation), focused on children or individual cancer sites, or without dosimetry. Further, nine publications were not included in our analysis mainly because relative risk estimates and their standard deviations could not be derived,^7^ ^8^ ^9^ ^10^ ^11^ because there were many cohort members with high exposures,^12^ ^13^ because no data of the Life Span Study (LSS) were available for the corresponding group of cancers among the atomic bomb survivors^14^ or the required information on age at exposure and age at risk were not obtained.^15^

If a study contained results for different cancer outcomes, then the outcome closest to “solid cancer” was chosen. Especially, inclusion of leukaemia was avoided as far as possible because of differences in height of risk and in shape of dose response, if compared with solid cancer.

Concerning the 15-countries collaborative study of cancer risk among radiation workers in the nuclear industry,^16^ the present analysis includes only results, which are not based on the Canadian data, because problems with the application of the Canadian data within the 15-countries study have been reported (Norman Gentner, personal communication, 2008).

### ERR per dose for atomic bomb survivors

The publicly available atomic bomb survivor datasets for cancer mortality from 1950 to 2000 (DS02can.dat) and cancer incidence from 1958 to 1998 (lssinci07.csv) from the Radiation Effects Research Foundation (http://www.rerf.or.jp) were used to calculate ERR-per-dose values for acute exposures. Only survivors with doses below 4 Gy of shielded kerma were used in the risk analysis.

The atomic bomb survivor data for the cancer categories used in an LDRMD study, *i* were fitted with a model including an explicit ERR-per-dose parameter, *β*_lss*i*_, a male fraction, *f_i_*, an age at exposure, *e_i_* and an age-attained, *a_i_*:

*λ*(*d_i_*, *s*, *e*, *a*)  =  *λ*_0_(*s, e, a*) [1+ *β*_lss*i*_ *d_i_* *ρ_i_*(*s*, *e*, *a*)] (1)

with

*ρ_i_*(*s*, *e*, *a*)  =  *θ_i_*(*s*) exp[*α_i_* (*e* − *e_i_*) + *ω_i_* ln (*a*/*a_i_*)] (2)

and

*θ_i_*(*s*)  =  1+ *θ_i_*_s_ *f_i_*, if *s*  =  female

(3)

*θ_i_*(*s*)  =  1− *θ_i_*_s_ (1− *f_i_*), if *s*  =  male

Here *λ* is the total mortality/incidence rate, *λ*_0_ the baseline rate, *d_i_* the dose (see below), *s* gender, *e* the age at exposure, *a* the age at risk and *α_i_,* *ω_i_,* and *θ_i_*_s_ are parameters. For *e_i_*, the average age at start of follow-up in the LDRMD study was chosen as a surrogate for average age at exposure. The modelling of age-at-exposure and age-attained dependences in equation (2) is the way the age parameters are treated in recent A-bomb papers, for example, by Preston *et al*.^17^

We based the risk calculations for the atomic bomb survivors

- on the dose to that organ as it was used in the corresponding LDRMD study, if the study was based on an organ dose
- the skin dose, if the LDRMD study was based on film badge or TLD readings.

Neutron doses were weighed by a factor of 10.

The Poisson regressions were performed with the programme AMFIT of the software package EPICURE (HiroSoft International Corp., Seattle, Washington, USA).

### Ratio of ERR-per-dose values

The ratio of the ERR-per-dose value, *β*_ldrmd*i*_, in an LDRMD study *i* and the corresponding value for the atomic bomb survivors was calculated as:

*q_i_*  =  *β*_ldrmd*i*_/*β*_lss*i*_. (4)

Normal distributions were assumed for *β*_ldrmd*i*_ and *β*_lss*i*_ with average values corresponding to the best estimates given in the publications (for *β*_ldrmd*i*_) or obtained in the Poisson regression (for *β*_lss*i*_). Standard deviations of the single estimates were estimated by dividing the width of their respective confidence interval by twice the appropriate quantile of the normal distribution. Percentiles and the variance *V_i_* of *q_i_* were calculated from 1000 samples from each distribution generated with the Monte Carlo software package Crystal Ball (Decisioneering, Denver, Colorado, USA).

### Combined estimator of the risk ratio

A combined estimator of the ratio of the ERR-per-dose values for LDRMD and acute exposures was obtained by the inverse variance method for calculating a weighted average of the ratios for the single LDRMD studies.


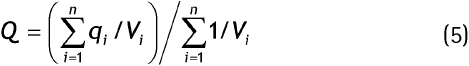


where n is the number of LDRMD studies considered.

The ratio *Q* was calculated separately for studies of cancer mortality and for studies of cancer incidence. Some of the LDRMD mortality studies had part of the data in common. In order to avoid a double counting of such mortality data, two analyses including only independent studies were performed. In the first analysis, LDRMD studies with the larger number of cancer mortality cases were used. In the second analysis, instead of these, LDRMD studies with the smaller number of cases were included. Out of the three analyses (two for cancer mortality and one for incidence) the combined risk estimator with the narrowest uncertainty range (the ratio of the upper and the lower boundary of the 90% confidence interval) was defined to be the main analysis. Sensitivity analyses were performed by excluding single studies from the main analysis. Study heterogeneity was assessed by calculating Cochran’s Q statistic and the corresponding p value.

### Lifetime risks

The BEIR VII committee performed a probabilistic calculation of the lifetime-solid-cancer mortality and incidence risks per dose for low-dose-rate exposures to external radiation according to:

*lr*_BEIRVII_  =  *lr*_lss_/*DDREF*_BEIRVII_ (6)

where *lr*_lss_ is the lifetime risk per dose for acute, high-dose exposures as derived for most cancer sites from the incidence data of the atomic bomb survivors from Hiroshima and Nagasaki, transferred to the American population.^3^ *DDREF*_BEIRVII_ has a mode of 1.5 and a 95% CI of 1.1 to 2.3.

Lifetime-solid-cancer mortality and incidence risks per dose for LDRMD exposures have been calculated here as:

*lr*_ldrmd_  =  *lr*_BEIRVII_ *DDREF*_BEIRVII_ *Q* (7)

In the calculation, *lr*_BEIRVII_ and *DDREF*_BEIRVII_ were assumed to be negatively correlated with a correlation coefficient of −0.5. In order to check the impact of this subjective choice, limiting calculations were also performed for values of the correlation coefficients of 0 and −1.

The ICRP has defined the detriment-adjusted nominal risk coefficient as a weighted sum of lifetime risks per dose for fatal and non-fatal cancer, severe heritable effects, and length of life lost. The coefficient is calculated by:

*d*_ICRP_  =  *d*_lss_/*DDREF*_ICRP_ (8)

where *d*_lss_ is the detriment-adjusted nominal risk coefficient for cancer after acute, high-dose exposures as derived mainly from the incidence data of the atomic bomb survivors.^2^ *DDREF*_ICRP_ has the value of 2.

Taking account of the cancer risk per dose in LDRMD epidemiological studies, a detriment-adjusted nominal risk coefficient for cancer was assessed here according to:

*d*_ldrmd_  =  *d*_ICRP_ *DDREF*_ICRP_ *Q* (9)

## Results

### Studies of low-dose-rate, moderate-dose exposures

All 12 studies selected for the analysis were cohort studies. The nine mortality studies (table 1) and three incidence studies (table 2) included seven studies on radiation workers,^5^ ^6^ ^16^ ^18^ ^19^ ^20^ three studies on emergency and clean-up workers after the Chernobyl accident^4^ ^21^ ^22^ and two studies on the residents of villages located along the banks of the Techa River.^23^ ^24^ Although a number of Chernobyl liquidators have obtained high-dose-rate exposures, the studies are included here, because the vast majority had only low-dose-rate exposures. None of the 12 studies include a considerable number of cohort members with cumulative exposures exceeding a few hundred milligray.

**Table 1:** List of cancer mortality studies, which were included in the analysis

No	Reference, country	Population	Follow-up, cancer cases	Type of exposure	Cancer outcomes	ERR per dose, *β*_ldrmd_ (Gy)^−1^, best estimate and 90% CI
1	Boice 2006, USA^18^	Workers at Rocketdyne	−1999, 3066	External and internal	All cancer excluding leukaemia	0.0 (−1.9 to 2.4)*
2	Cardis 2007, 14 countries†^16^	Radiation workers in nuclear industry	Variable, 6119	External	All cancer excluding leukaemia	0.6 (−0.1 to 1.4)
3	Ivanov 2001, Russia‡^21^	Chernobyl clean-up workers	1991–1998, 515	External	Neoplasms ICD-9 140–239	2.1 (1.3 to 2.9)*
4	Ivanov 2006, Russia^4^	Chernobyl clean-up workers	1992–2002, 651	External	Solid cancer	1.5 (0.2 to 2.9)*
5	Krestinina 2005, Russia^24^	Techa River residents	−1999, 1842	External and internal	Solid cancer except bone cancer	0.9 (0.2 to 1.7)*
6	Muirhead 2009, UK^6^	Radiation workers	−2001, 6959	External	Malignant neoplasms excluding leukaemia	0.3 (0.02 to 0.6)
7	Stayner 2007, USA^5^	ORNL workers	−1984, 225	External	All cancer excluding leukaemia	4.8 (0.4 to 13.3)§
8	Telle-Lamberton 2007, France¶^19^	French nuclear workers	1968–1994, 721	External	All cancer excluding leukaemia	1.5 (−0.5 to 4.0)
9	Wing 2005, USA^20^	Hanford workers	−1994, 2265	External and internal	All cancer	0.3 (−0.3 to 1.0)

ERR, excess relative risk; ORNL, Oak Ridge National Laboratory.

*95% confidence interval.

†Canadian data excluded from 15-countries study.

‡Summarised by Ivanov *et al*^25^ in 2007, and therefore included in our analysis.

§After correction for dose uncertainties. The result without this correction is 5.4 (0.5 to 12.6) Gy^−1^.

¶Results for “all cancer excluding leukaemia” were supplied by personal communication with Telle-Lamberton (2008).

**Table 2:** List of cancer incidence studies, which were included in the analysis

No	Reference, country	Population	Follow-up, cancer cases	Type of exposure	Cancer outcomes	ERR per dose, *β*_ldrmd_ (Gy)^−1^, best estimate and 90% CI
10	Ivanov 2004, Russia^22^	Chernobyl clean-up workers	1996–2001, 1370	External	Solid cancer	0.3 (−0.4 to 1.2)*
11	Krestinina 2007, Russia^23^	Techa River residents	1956–2002, 1836	External and internal	Solid cancer except bone cancer	1.0 (0.3 to 1.9)*
12	Muirhead 2009, UK^6^	Radiation workers	−2001, 10 855	External	Malignant neoplasms excluding leukaemia	0.3 (0.04 to 0.5)

*95% confidence interval.

The best estimates of the ERR were positive in all studies (in one study it was 0.0). In seven of the 12 studies the excess cancer risk was significantly related to the radiation exposure.

### ERR per dose for atomic bomb survivors

The ERR-per-dose estimates for the atomic bomb survivors matched by categories of cancer mortality, sex ratios, average ages at exposure and average ages at risk of the LDRMD studies vary by more than a factor of 2.5 (tables 3 and 4). The highest estimate corresponds to the conditions in the cancer incidence study of the Techa River residents: a value of 0.59 (95% CI 0.49 to 0.69) Gy^−1^ is obtained for relatively young average age at first exposure (25 years) and a large fraction of females (0.57). Also, the risk estimation is related to the dose in a relatively well-shielded organ (stomach). The lowest estimate corresponds to a mortality study of Chernobyl liquidators: a value of 0.23 (95% CI 0.11 to 0.34) Gy^−1^ is obtained for all solid cancer and a high male fraction (100% males). Further, the risk is related to the relatively high dose in skin.

**Table 3:** Datasets, parameters and risk per dose for the atomic bomb survivors corresponding to the low-dose-rate, moderate-dose studies of cancer mortality in table 1, and the risk ratios, *q_i_*

No	Population	Male fraction	Average age at start of follow-up	Average age at end of follow-up	Dose quantity	ERR per dose (Gy^−1^) in LSS, *β*_lss_, best estimate and 90% CI	Risk ratio, *q*, best estimate and 90% CI
1	Workers at Rocketdyne	0.92	31	56	Skin dose	0.26 (0.16 to 0.35)*	0.00 (−7.25 to 7.33)
2	Radiation workers in nuclear industry	0.90	31	46	Colon dose	0.49 (0.30 to 0.67)	1.19 (−0.34 to 3.12)
3	Chernobyl clean-up workers†	1.00	35	47	Skin dose	0.47 (0.29 to 0.65)*	4.49 (2.79 to 7.17)
4	Chernobyl clean-up workers	1.00	35	50	Skin dose	0.23 (0.11 to 0.34)*	6.66 (1.67 to 14.7)
5	Techa River residents‡	0.40	28	63	Stomach dose	0.54 (0.42 to 0.65)*	1.71 (0.52 to 3.04)
6	UK radiation workers	0.90	29	52	Skin dose	0.30 (0.21 to 0.39)	0.91 (0.01 to 2.01)
7	ORNL workers	1.00	30	57	Skin dose	0.25 (0.16 to 0.33)	19.6 (−6.38 to 51.3)
8	French nuclear workers	0.79	31	49	Skin dose	0.33 (0.23 to 0.43)	4.59 (−2.34 to 12.6)
9	Hanford workers	0.76	31	55	Skin dose	0.41 (0.33 to 0.49)	0.73 (−0.87 to 2.35)

ORNL, Oak Ridge National Laboratory.

*95% confidence interval.

†Calculations performed for all cancer, because out of 515 neoplasms (ICD-9 140–239) there were only three non-cancer cases (ICD-9 208–239).

‡Calculations performed for all solid cancer, because mortality data with DS02 were not available for bone cancer.

**Table 4:** Datasets, parameters and risk per dose for the atomic bomb survivors corresponding to the low-dose-rate, moderate-dose studies of cancer incidence in table 2, and the risk ratios, *q_i_*

No	Population	Male fraction	Average age at start of follow-up	Average age at end of follow-up	Dose quantity	ERR per dose (Gy^−1^) for acute exposure, *β*_lss_, best estimate and 90% CI	Risk ratio, *q*, best estimate and 90% CI
10	Chernobyl clean-up workers	1.00	35	49	Skin dose	0.33 (0.21 to 0.46)*	0.99 (−1.10 to 3.25)
11	Techa River residents	0.43	25	65	Stomach dose	0.59 (0.49 to 0.69)*	1.70 (0.54 to 2.92)
12	UK radiation workers	0.90	29	52	Skin dose	0.37 (0.29 to 0.46)	0.71 (0.09 to 1.42)

*95% confidence interval.

### Comparison of ERR-per-dose values for different types of exposure

**Figure 1:**
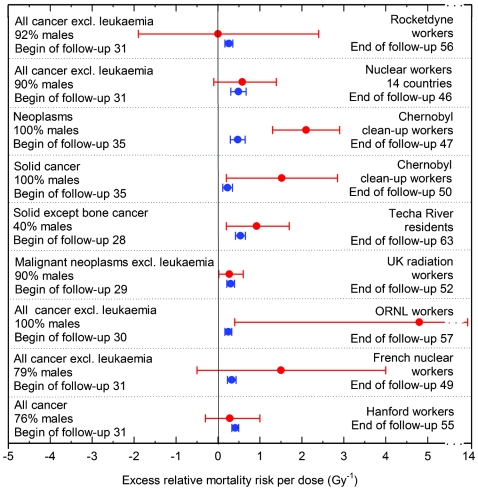
Excess relative risk per dose for cancer mortality in nine studies of low-dose-rate, moderate-dose exposures (red symbols), as compared with acute, high-dose exposures (atomic bomb survivors of Hiroshima and Nagasaki) (blue symbols). The error bars indicate 95% CIs for the studies of workers at Rocketdyne, the Chernobyl emergency workers and the Techa River residents, and 90% CIs for all other studies.

**Figure 2:**
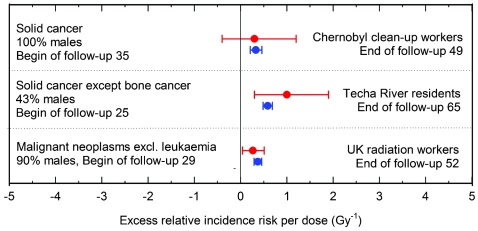
Excess relative risk per dose for cancer incidence in three studies of low-dose-rate, moderate-dose exposures (red symbols), as compared with acute, high-dose exposures (atomic bomb survivors of Hiroshima and Nagasaki) (blue symbols). The error bars indicate 95% CIs for the Chernobyl emergency workers and the Techa River residents, and 90% CIs for the UK National Registry for Radiation Workers.

Generally, the uncertainties of the ERR estimates in the LDRMD studies are much larger than the corresponding estimates for atomic bomb survivors (figs 1 and 2). In six of the 12 LDRMD studies, the best estimate of the ERR per dose is larger than that for the atomic bomb survivors by more than a factor of 1.5, in five studies it is comparable, and only in one study it is smaller by more than a factor of 1.5.

The risk ratio, *q*, is significantly larger than 1.0 for the two mortality studies of Chernobyl clean-up workers.^4^ ^21^ In the remaining 10 LDRMD studies, the cancer-risk-per-dose values are compatible with those from the study of the atomic bomb survivors.

### Combined estimator of the risk ratio

No statistical heterogeneity was detected between the estimated ratios, *q_i_*, included in each of the three analyses (table 5). It should be noted, however, that the power of the test is not strong in view of the small number of studies included. The uncertainty range of the combined estimator for the larger mortality studies and for the incidence studies had the same width. The analysis of the larger mortality studies was chosen as the main analysis because it includes more studies.

**Table 5:** Ratios of the excess relative risk per dose in low-dose-rate, moderate-dose studies and for the atomic bomb survivors as calculated in three analyses (main analysis in bold)

Endpoint	Criterion to select independent studies	Numbers of studies included*	Risk ratio, *Q*, best estimate and 90% CI	p Value for heterogeneity
**Mortality**	**Larger number of cancer cases**	**1, 4, 5, 6, 7, 8, 9**	**1.21 (0.51 to 1.90)**	**0.79**
Mortality	Smaller number of cancer cases	1, 2, 3, 5	2.08 (1.16 to 3.01)	0.21
Incidence	–	10, 11, 12	0.98 (0.41 to 1.54)	0.49

*Compare tables 1 and 2.

The main analysis includes seven cancer mortality studies, five of nuclear workers,^5^ ^6^ ^18^ ^19^ ^20^ one of Chernobyl emergency and clean-up workers^21^ and one of Techa River residents.^24^ A risk ratio, *Q*, of 1.21 (90% CI 0.51 to 1.90) is obtained. The best estimate for the smaller mortality studies is larger; the difference is, however, not significant (p = 0.16). The combined estimator for the incidence studies is relatively close to the result of the main analysis.

Leaving out one of the studies changed the best estimate of *Q* in the main analysis at most by 26%. The lowest risk ratio with a value of 0.96 (90% CI 0.12 to 1.80) was obtained when the study of the Techa River residents was excluded. The highest risk ratio with a value of 1.44 (90% CI 0.48 to 2.41) was obtained when the study of the UK radiation workers was excluded.

### Lifetime risks

Based on assessments of BEIR VII for lifetime cancer risks after acute exposures and on the results of the present analysis (equation 7), a number of about 14 (90% CI 6 to 31) or 24 (90% CI 9 to 49) excess solid cancer cases among 1000 males or females, respectively, is obtained for LDRMD gamma-ray exposures with a dose of 100 mGy. It is further estimated that there would be about seven (90% CI 3 to 15) or 11 (90% CI 4 to 23) excess fatalities from solid cancer among males or females, respectively.

If *lr*_BEIRVII_ and *DDREF*_BEIRVII_ were assumed to be not or completely anti-correlated, then the best estimates of the lifetime risks are essentially the same and the confidence intervals are increased or decreased by about 30%, respectively.

The radiation protection system of the ICRP is based on the effective dose. For whole body exposures with low-LET radiation, the effective dose in the unit Sievert (Sv) is numerically equal to the absorbed dose in the unit Gray (Gy) as it was used by BEIR VII. Based on the assessment of the ICRP for the detriment-adjusted nominal cancer risk coefficient for acute exposures and on the result of the present analysis (equation 9), an estimate of the detriment-adjusted nominal risk coefficient for workers of about 10 (90% CI 4 to 16) 10^−2^ Sv^−1^ is obtained for LDRMD exposures. Representing essentially a sum of excess cancer fatalities and of weighted excess non-fatal cancer cases, this value is slightly larger than the sex-averaged result for the mortality risk as described above.

## Discussion

### Strengths and limitations of the present study

It is the strength of the analysis to have extracted the following common information from a number of recent epidemiological studies of cancer after LDRMD exposures:

- There is evidence for an excess cancer risk after LDRMD exposures to ionising radiation.
- There is no indication that the excess cancer risk per dose for LDRMD exposures is smaller than for the atomic bomb survivors.
- These results still hold if single studies are excluded from the analysis.

Most of the studies included in the present analysis have methodological limitations especially concerning dosimetry. It is impossible to predict how improvements of dosimetry would or will change the results of the single LDRMD studies. A Monte Carlo simulation study incorporating uncertainty in the dose parameters estimated for study of ORNL workers found very little impact of these uncertainties on ERR-per-dose estimates.^5^ Further, if future changes of the results of several LDRMD studies do not go in the same direction (increasing or decreasing the risk), then implications for the general results of the present analyses are expected to be low, because

- the risk ratios in the three different analyses presented in table 5 are quite consistent;
- the risk ratio of the main analysis is not strongly affected by a single study.

Another severe limitation of the LDRMD studies is the non-availability of data on risk factors other then radiation, especially of smoking data. Such risk factors may confound the results. Since, however, neither the LDRMD studies nor the analyses of the atomic bomb survivors take such risk factors into account, the risk ratios derived in the present paper may be less affected by the missing information than the risk estimates themselves.

A main limitation of the present analysis is the inclusion of results for different exposed groups and different groups of cancer types. Indeed, the relative risks among the atomic bomb survivors matching the conditions of the LDRMD studies vary by more than a factor of 2.5. There is no obvious way to avoid this limitation because the available single studies and even the large 15-countries pooled analysis do not have enough statistical power to allow conclusions as drawn in the present paper. However, the calculation of risk ratios for comparable conditions (groups of cancer types, male fraction, age at exposure, age attained, dose quantity used in the risk analysis, mortality or incidence data) in the present paper and the determination of a combined estimator for these ratios alleviate the problem with heterogeneous study conditions and endpoints.

Another limitation is the fact that published risk estimates were used instead of individual data from the included studies. Access to individual data from some of the excluded studies is possible via the Comprehensive Epidemiologic Data Resource (http://cedr.lbl.gov/). However, for the current analyses such extensive data acquisition and analysis could not be undertaken.

Finally, in the comparison of risks from protracted and acute exposures, the definition of age at exposure is problematic. In the present analysis, the average age at the start of follow-up has been used in the comparison. An older effective age at exposure would be more correct, but could not be estimated in this study. Using an older effective age at exposure would result in lower ERR-per-dose estimates for acute exposures and thus in even higher *q_i_* values than obtained in the present analysis.

In summary, the value of the present study is a general estimation of implications of published studies rather than a quantitative risk evaluation.

### Comparison with low-dose-rate, high-dose exposures

Two papers have been published in the past few years on large cohort studies of solid cancer risk due to low-dose-rate, but high-dose exposures.

One study included workers at the Mayak Production Association in the Southern Urals, Russia, which produced plutonium for the atomic weapons of the former Soviet Union.^13^ These workers were exposed to external radiation and to plutonium which exposed mainly lungs, liver and bone. A first analysis of the cancer mortality with regard to other organs yielded an estimate of the ERR per external dose which was considerably lower than that for the atomic bomb survivors. It may, however, be noted that leukaemia risks per dose were quite comparable.

The second study included residents of northern Kazakhstan who were exposed to the fallout and also to external radiation from atomic bomb explosions performed at the nuclear Semipalatinsk test site.^12^ The best estimate of the excess relative cancer mortality risk per dose was considerably higher than that for the atomic bomb survivors.

In summary, these high-dose studies do not provide contradictory evidence for the present evaluation of LDRMD exposure studies.

### Comparison with BEIR VII and ICRP recommendations

According to BEIR VII, cancer risk after LDRMD exposure is expected to be by a factor of 1.5, according to the ICRP by a factor of 2, smaller than among atomic bomb survivors. However, the best estimates of the cancer risk in 11 of the 12 LDRMD studies are larger than both expectations (tables 3 and 4).

Due to low statistical power most single studies are consistent with the BEIR VII and ICRP recommendations: the 90% confidence ranges of 10 of the 12 risk ratios, *q*_i_, include the value of 0.67, corresponding to the inverse *DDREF* value used by BEIR VII; eight include the ICRP value of 0.5.

**Figure 3:**
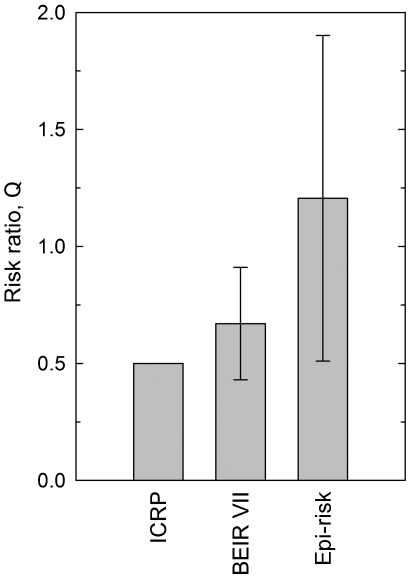
Ratio *Q* of excess relative risk-per-dose values for cancer after low-dose-rate, moderate-dose exposures and after acute, high-dose exposures as recommended by the International Commission on Radiological Protection (ICRP),^2^ used by BEIR VII (95% CI),^3^ and derived in the present analysis from epidemiological studies (epi-risk, 90% CI).

According to the main analysis in the present paper, the combined estimator of the risk ratio, *Q*, is compatible with the *DDREF* used in BEIR VII, although the BEIR VII risk estimates are in the lower range (fig 3). The risk value recommended by the ICRP is smaller than the present result for LDRMD exposures. This result is borderline significant on the 90% confidence level.

The ICRP and BEIR VII base their *DDREF*s mainly on radiobiological results including animal data, which, in their majority, suggest a characteristically low risk for low-dose-rate exposures. It remains an open question as to why this characteristic is apparently not reflected in the human epidemiological data.

### Implications

The recent epidemiological studies analysed here provide some evidence that cancer risks associated with LDRMD exposures to ionising radiation may be greater than those published by BEIR VII and the ICRP.

The ICRP rationale for radiation protection is based on three concepts: justification, dose limitation, and optimisation. The results of the new epidemiological studies highlight the need for justification of the use of radionuclides and ionising radiation in medicine, industry and research. Derivation of dose limits for radiation protection is a complex process including, for example, comparisons of occupational exposures with exposures to radiation from natural sources, or of radiation risks with other occupational health and mortality risks. Compared with earlier recommendations, the ICRP decided in 1991 to considerably reduce the recommended limit on effective dose for occupational exposures to 20 mSv per year, averaged over 5 years (100 mSv in 5 years).^26^ Estimates of cancer risks related to exposures with cumulated doses of 100 mSv have been given in the Results section.

The ICRP has defined optimisation “as the source-related process to keep the likelihood of incurring exposures…, the number of people exposed, and the magnitude of individual doses as low as reasonably achievable, taking economic and societal factors into account”.^2^ The new epidemiological results may influence optimisation procedures for future use of radionuclides and ionising radiation.

Probability-of-causation calculations play an important role in the adjudication of claims of compensations for cancer diseases after occupational radiation exposures. The computer code IREP made available by the US National Institute for Occupational Safety and Health (http://www.niosh-irep.com) is widely used for these calculations. The IREP includes a DDREF, which lowers the probability of causation for low-dose-rate exposures.^27^ Use of such a factor in these calculations is questioned by the new epidemiological studies. Indeed, in the UK compensation scheme it is not assumed that low-dose exposures result in a lower risk per dose than acute, high-dose exposures.^28^

## Appendix 1: Relation of whole body dose and skin dose

The term “whole body dose”, as used in a number of epidemiological studies of workers exposed to ionising radiation, relates to the dosimeter dose worn in front of the trunk of the worker. Values for this dose quantity are not available for the atomic bomb survivors. The main exposure of the atomic bomb survivors is due to Roentgen rays or gamma rays in the energy range of 100 keV to a few MeV. In this Appendix an organ is identified, for which dose values are available and which may serve as a surrogate for the whole body dose among atomic bomb survivors.

Zankl published conversion coefficients for the whole body dose, or more specifically for the personal dose equivalent, H_p_(10), per air kerma free in air, K_a_ (in Sv Gy^−1^) for a typical dosimeter position, for monoenergetic photons incident in various irradiation geometries.^29^ The ICRP published conversion coefficients of 15 organs in an anthropomorphic phantom per kerma free in air for monoenergetic photons incident in various irradiation geometries.^30^

We calculated the ratios of these two sets of conversion factors for two irradiation geometries: parallel from the front (anterior-posterior) and parallel from all horizontal directions (rotational invariant). For the photon energies and irradiation geometries of interest, the conversion coefficients for skin were found to be similar to the conversion coefficients for whole body dose: for both irradiation geometries and the whole energy range the coefficients agree within 10%.
